# Bioinspired Slippery Lubricant-Infused Surfaces With External Stimuli Responsive Wettability: A Mini Review

**DOI:** 10.3389/fchem.2019.00826

**Published:** 2019-11-29

**Authors:** Xian Yang, Yu Huang, Yan Zhao, Xiaoyu Zhang, Jinhua Wang, Ei Ei Sann, Khin Hla Mon, Xiaoding Lou, Fan Xia

**Affiliations:** ^1^Engineering Research Center of Nano-Geomaterials of Ministry of Education, Faculty of Material Science and Chemistry, China University of Geosciences, Wuhan, China; ^2^Department of Materials Science, Institute of Molecular Materials and Devices, Fudan University, Shanghai, China; ^3^Department of Industrial Chemistry, Dagon University, Yangon, Myanmar; ^4^Key Laboratory of Bio-inspired Materials and Interfacial Science, Technical Institute of Physics and Chemistry, Chinese Academy of Science, Beijing, China

**Keywords:** bio-inspired, liquid-infused surfaces, slippery surface, stimuli response, wettability, interfacial adhesion

## Abstract

Responsive slippery lubricant-infused surfaces (SLIS) have attracted substantial attention because of the high demand of fundamental research and practical applications, such as controllable liquid-repellency, intelligent, and easy-to-implement wettability switching. In this review, advanced development of responsive slippery surfaces is briefly summarized upon various external stimuli, including stress, electrical field, magnetic field, and temperature. In addition, remaining challenge and prospect are also discussed.

## Introduction

**Figure 1:**
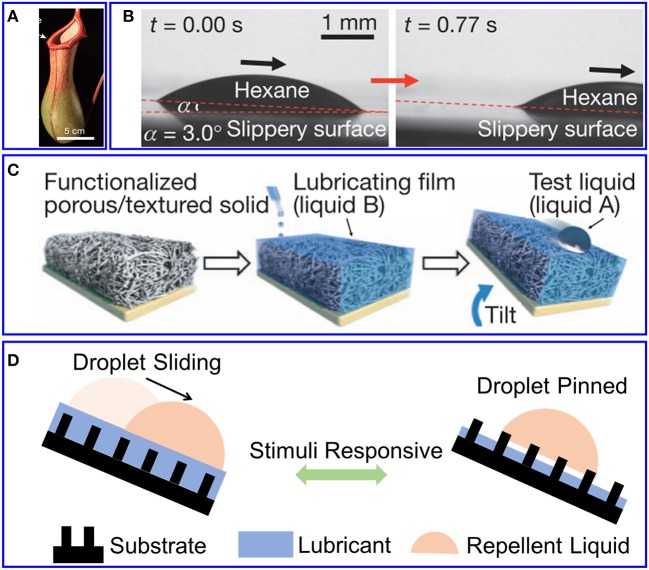
SLIS inspired by pitch plant. **(A)** Photo of pitch plant (Chen et al., 2016). Reproduced with permission. Copyright 2016, Springer Nature. **(B)** A droplet of hexane sliding on SLIS (Wong et al., 2011). Reproduced with permission. Copyright 2011, Springer Nature. **(C)** Schematic fabrication of SLIS (Wong et al., 2011). Reproduced with permission. Copyright 2011, Springer Nature. **(D)** Schematic illustration of stimuli responsive SLIS.

Surface's wettability is one of the most fundamental performances in numerous biological processes and industrial technologies, which attracts researchers' interest for a long time. In the past decades, owing to new understanding of species wetting mechanism, bio-inspired materials with super-wettability are flourishing through modeled after surface morphology and chemical composition of the nature species (Liu et al., 2017; Sett et al., 2017; Li et al., 2019). For example, *Nepenthes* pitcher plants fill water in the spaces among the micro structure to form a slippery liquid film, leading insects to aquaplane into their stomach. Since Wong et al. (2011) mimicked pitcher plants' slippery surface and fabricated slippery liquid-infused porous surfaces (Figures 1A,B), a serial of liquid-infused surfaces, or well-known as slippery lubricant-infused surfaces (SLIS), have been developed for various applications, such as liquid repellency (Hozumi et al., 2011; Huang et al., 2017), liquid or gas transportation (Chen et al., 2016; Xiao et al., 2019), water harvesting (Zhang et al., 2017), oil-water separation (Solomon et al., 2014; Wang et al., 2017a), anti-corrosion (Lee et al., 2017; Howell et al., 2018), heat transfer (Anand et al., 2012), visual biosensors (Gao et al., 2018), and so on. The two basic components of SLIS are solid substrates to hold liquid and the liquid for infiltration, as shown in Figure 1C. The solid substrates may have a micro/nano structural surface to infuse lubricant (Xiao et al., 2013; Dai et al., 2015), or can be swollen in lubricant (Yao et al., 2014). The liquid hold by the solid substrates acts as stable lubricant to repel any impinging immiscible fluids, and ensures the repellent fluids to slide away without any resistance. To distinguish the liquid for infused and the fluids for repellency, we refer the infused liquid as “lubricant” in this mini review from now on. No matter the substrates' surfaces are rough or flat, the key feature of SLIS is the thin layer of lubricant cover the substrate. This lubricant layer forms a dynamic and stable lubricant/substrate interface. If this thin lubricant is removed, the film would lose its excellent liquid repellency and droplet pin on the surface as a result. This is because the interfacial adhesion of the repelling liquid/solid surface is obviously larger than that of the repelling liquid/lubricant, as shown in Figure 1D. Based on this mechanism, SLIS with controllable and reversible wettability were developed through stimulus-triggered switch in substrate's chemical composition or/and morphologies, lubricant's height or/and phase, repellent liquid's chemical component and so on. Meanwhile, other properties, like optical property, will also change and endow SLIS with different functionalities. The responsive SLIS have demonstrated their ability of controllable repellent droplet's motion behavior, which provides significant insight of developing devices for fog collection (Peng et al., 2015), oil-water separation (Calcagnile et al., 2012), droplets' delivery (Hou et al., 2017), complex-flow distribution (Cao et al., 2017), biomimetic tissue (Wang Y. et al., 2018), and so on.

In addition, typical response materials have been adopted for different external stimuli. Elastic and nanoporous polymers such as polydimethylsiloxane (PDMS) and polyurethane (PU) are mainstream in mechanically responsive SLIS. Electrical responsive materials are chosen primarily based on electrowetting mechanism, generally both the substrate and the repellent liquid should be conductive. Magnetic field response strategies include magnetic particles doped substrates or introduces magnetic lubricant. Traditional thermal response is mainly realized by thermosensitive lubricant such as paraffin or mixtures of solid/liquid oil.

## External Stimuli Responsive SLIS

### Mechanically Responsive SLIS

**Figure 2:**
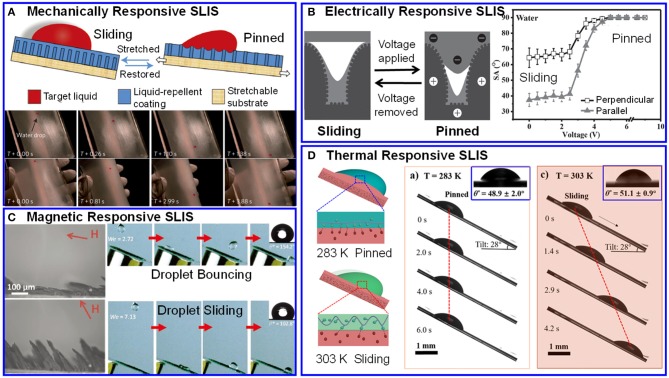
Responsive SLIS display different wetting behaviors by different stimulus triggered. **(A)** Stress responsive SLIS (Yao et al., 2013), Reproduced with permission. Copyright 2013, Springer Nature. **(B)** Electric field responsive SLIS (Guo et al., 2016), Reproduced with permission. Copyright 2016, WILEY-VCH Verlag GmbH & Co. KGaA. **(C)** Magnetic field responsive SLIS (Huang et al., 2017), Copyright 2017, WILEY-VCH Verlag GmbH & Co. KGaA. **(D)** Temperature responsive SLIS (Wang et al., 2019). Copyright 2019, ACS.

Stretching or mechanical pressing represents a simple, straightforward and effective strategy for tuning the wettability of SLIS. Generally, replacing the rigid substrate with a continuous elastic polymer is required to enable mechanical deformation. The stress would vary the surface's morphology and lubricant layer's height, resulting in the change of surface's wettability. Yao et al. (2013) adopted a nanoporous elastic PDMS substrate to support lubricant. This film would reversibly adjust the droplet's sliding behaviors on it, as shown in Figure 2A. When the film was stretched, the lubricant would flow into the pores. In this case, the solid surface formed a rough interface, resulting in the droplet pinned. Without stress, the lubricant came out from the porous. The film restored its liquid repellency and made the droplet slide away. In this research, graded mechanical stimulus was proved to lead to dynamic and precise regulation of optical transparency and wettability. This film provided a new idea for fuel transport pipes or microfluidic systems. Based on Yao's research, Liu et al. (2016) constructed an elastic substrate with periodical porous structure instead of atactic porous. The film was demonstrated a variable structural-color SLIS with self-reporting surface wettability. When the film was elastically deformed by stress, lubricant thickness and structural color of surface would change simultaneously along the deformation of porous. Consequently, the accompaniment of these changes made self-reporting of surface wettability a reality. Beside optical devices, SLIS with controllable wettability also demonstrated their applications in collecting water from atmosphere or water-oil mixture (Han et al., 2016; Park et al., 2016; Fu et al., 2017). Most research was focused on strengthening condensation and shedding of water, avoiding the wind-caused loss of the water captured. Wang et al. (2017b) developed a flexible SLIS by infusing perfluoropolyether into a fluorinated-copolymer-modified PU with adjustable and elastic deformability. Through controlling the droplets' sliding behavior, the stress responsive SLIS showed some adaptability of environmental and realized high efficiency of water collection.

Although the mechanical responsive SLIS have been demonstrated reversible deformation and wettability switching, their dependence on elastic substrate severally limit their practical applications. What's more, continuous stress and deformation would trigger the potential mechanical damage. Improving the inherent durability of materials or introducing self-repairing materials should be a key point for developing mechanical responsive SLIS.

### Electrical Field Responsive SLIS

Electrowetting is a method of changing interfacial wettability by adjusting the potential between the liquid and solid electrodes. Due to its fast response, wide range changes, small size and good reliability, electrowetting method has been widely used in optics, biology, and microelectronics (Mugele and Baret, 2005). Generally, to achieve electrical field responsive SLIS, both the substrate and the repellent liquid should be conductive. Heng et al. firstly put forward a serial of SLIS with anisotropic and electrically responsive sliding (Guo et al., 2016; Che et al., 2017; Wang et al., 2018a,b; Han et al., 2019). Firstly, by directional freeze drying, they synthesized oriented and conductive membrane with porous structure. Then, silicone oil was introduced into the membrane. With voltage applied, the surface's chemical components and micro/nano structure won't change. However, electrostatic attraction between the repellent conductive droplet and its image charge increased under voltage. The thickness of the lubricant reduced, and droplet's wetting status changed. As a result, the droplet was pinned on the surface (Figure 2B). In the other hand, without voltage, the conductive droplet slid easily due to the thick layer of lubricant. Later on, Heng's group (Che et al., 2017) filled the surface with conductive lubricant. Compared with non-conductive lubricant, an electrical double layer with higher charge density was achieved with the same voltage. Therefore, the SLIS realized droplet's reversible motion control with a smaller voltage. They (Wang et al., 2018a) further researched the lubricant's viscosity effected on the electrical field responsive SLIS. The results suggested that lubricant with low viscosity would require a smaller voltage to adjust the droplet's motion behavior. Based on tuning the properties of lubricant, Heng's a serial of research provided novel insights of SLIS' wettability adjustment. In addition, they (Wang et al., 2018b) also developed a photoelectric synergetic responsive SLIS. With voltage applied in dark, there was an electrical double layer. With illumination, the photo-generated electrons would increase the charge density, which results in a larger friction force to pin the droplet on the surface.

To achieve electrically responsive SLIS, relevant chemical environment is fatally necessary. For example, the repellent liquid should always contact with an electrode. The preconditions severely limit their applications in conductive surface, conductive repellent liquid, and even conductive lubricant. Therefore, there is still a long way for electrically responsive SLIS to come to real-life applications.

### Magnetic Field Responsive SLIS

Magnetic field is one of the most employed methods of adjusting surface's wettability. Compared with other physical stimuli, the structure of surface can switch in a short time driven by magnetic force. Meantime, applied magnetic fields with different directions provide more possibilities for changing surface's different properties. The magnetic field responsive SLIS with switchable wettability have achieved fruitful development in fog collection (Peng et al., 2015), oil-water separation (Calcagnile et al., 2012), complex-flow distribution (Cao et al., 2017), droplets' delivery (Hou et al., 2017), and so on.

For example, Cao et al. (2017) constructed ferromagnetic microcilia with different tilted angles to realize unidirectional wetting behaviors. On this film, droplets slid easily in the opposite direction of the microcilia's tilted, due to the low the liquid-air interfacial adhesion. Later on, they further developed a magnetically responsive surface to control water-droplet from rolling to pinning (Hou et al., 2017). Distinct from Cao's surface, our group (Huang et al., 2017) designed a magnetically dynamic surface inspired by pitcher plant and lotus leaf, which could reversibly switch droplet between SLIS state and superhydrophobic state. In this research, PDMS was mixed with iron powder to form magnetically responsive micropillar array. The micropillar array could transform from fully up-right (superhydrophobic state) to nearly flattened (SLIS state) morphology in different directions of external magnetic field, as shown in Figure 2C. The SLIS could alter liquid's repellency according to demand, which provided new opportunities of programmable fluid collection, smart waterproof clothing, adaptive drag control, and so on.

Instead of doping magnetic particles into the substrate, Tian et al. (2016) adopted magnetic fluids as lubricant and filled it into nano-structural substrate. The film's wettability could be adjusted by morphological transformation between rough and smooth in various magnetic fields, which provides new idea for designing microfluidic devices. Through magnetic lubricant, the tuning of interface's morphology could be more precise. Recently, Wang W. et al. (2018) designed a hierarchical magnetic responsive SLIS through infiltrating ferrofluids into surface with regular porous. The exiting capillary pressure induced multi-scale topographical responses and other novel functions. For example, when applied magnetic field, ferrofluid depleted from the microstructures. As a result, the non-magnetic colloidal particles fell down to the solid/lubricant interface and formed specific patterns. These multi-scale reconfigurable topographies can be used as biological tissues, responsive coating, and digital microfluidics.

Although various magnetic responsive SLIS have been demonstrated fast, controllable and flexible wettability switching, magnetic responsive substrates with large-scale and well-organized micro/nano structure are still rare. Combined with lithography technology may take into consideration.

### Temperature Responsive SLIS

Temperature is considered as a controllable and quantitative external stimulus, which extends widely applications *in vivo*, industrial and medical fields. Generally, for typical temperature responsive SLIS, thermo-responsive solidifiable lubricants were adopted to achieve interfacial adhesion switching (Yao et al., 2014; Manabe et al., 2016; Wang B. L. et al., 2018). In Yao et al. (2014), firstly introduced *n*-paraffin to organogel and form temperature responsive SLIS. In this work, switching of droplet's wettability occurred at the paraffin's melting temperature (*T*_*m*_). When the environmental temperature was higher than *T*_*m*_, paraffin was in the liquid phase, acting as lubricant filled in the PDMS network. Therefore, a droplet on SLIS was in low adhesive state. However, when the temperature dropped to lower than *T*_*m*_, paraffin turned into solid phase and became a part of solid substrate. The droplet on this surface was in high adhesive Wenzel state. Later on, they (Wang B. L. et al., 2018) combined anisotropic substrate and thermo-responsive SLIS to achieve more precise control of the liquid droplet motion. According to Yao's research, Manabe et al. (2016) adopted mixtures of solid/liquid paraffin as lubricant. Through adjusting the ratios of solid/liquid paraffin and ambient temperature, the film's transparency and wettability would change. Combined with optical property's change, the film displayed potential of applications in smart windows, innovative medicine, and other bio-chemical devices. Similarly, the change of lubricant condensed phase between liquid and gel also varied surface's wetting behaviors. Zhu et al. (2016) impregnated a nanostructured surface with heated mineral oil to fabricate temperature responsive SLIS. When the mineral oil cooled down to ambient temperature, the oil turned to gel. In this case, the interfacial adhesion would increase. By taking advantage of dynamic viscoelastic property of mineral oil, the droplet motion, sliding speed, and thermal variation can be well-controlled. Zheng et al. (2017) infused lubricant with lower critical solution temperature (LCST) to porous substrate. When the environmental temperature was higher than LCST (i.e., 313 K), water droplet was slippery on the surface. In contrast, when the temperature was lower than LCST (i.e., 293 K), water droplet was miscible with the lubricant. In their research, *in situ* wetting, dewetting, penetration and optical properties could be controlled under thermo-stimuli. Recently, combined printing method, Yao's group (He et al., 2018) adopted thermochromic inks to fabricate patterned thermo-responsive SLIS with multi-functionalities, including self-reporting wettability, and sensing the temperature of contacting liquids. This kind of slippery surface would be of great importance in sensors and medical package.

Generally, traditional smart SLIS focused on the responsive components contained in solid substrate and lubricant to vary the interface's topography, modulus, and surface energy. Modification of the repellent liquid was still rare. Recently, our group (Gao et al., 2018) approved that through adjusting the hydrophobic interactions between biological droplet and lubricant, the biological droplet's motion behavior could be easily tuned. The hydrophobic interactions mainly depended on the chain length of ssDNA in repellent droplet. To the best of our knowledge, this was the first research focused on adjusting the SLIS's interfacial adhesion through the repellent liquid. Their biosensing applications for ATP, microRNA, and thrombin detection are also demonstrated. Based on this research, very recently, we (Wang et al., 2019) put forward a temperature responsive interfacial adhesion on SLIS. When environmental temperature increased, ssDNA became more flexible and more mobile. The molecular configuration transformed to reduce the hydrophobic moieties exposure. As a result, the hydrophobic interaction between lubricant and hydrophobic moieties was weakened, leading to droplet slide, as shown in Figure 2D. The thermo-responsive sliding behavior of the biological droplet would offer a new strategy for advanced antifouling systems.

Although various temperature-responsive SLIS have been demonstrated, most of them are still restricted to several kinds of thermo-sensitive materials. Novel materials displaying obvious difference in wettability under small change of temperature should be explored. Additionally, besides temperature-dependent substrate and lubricant, thermo-responsive repellent liquid should be considered for particular applications, such as biological droplets in micro-biochips.

## Conclusion

In this review, we concentrate on recent development of SLIS with external stimuli responsive wettability, including stress responsive SLIS, electrical responsive SLIS, magnetically responsive SLIS, and thermo-responsive SLIS. Through introducing stimuli responsive materials or replace some parts of SLIS, repellent droplet's wetting behaviors and other properties of the film, such as structural colors and transparency, would be reversibly changed. Based on the responsive SLIS's ability of timely control of droplets, SLIS have demonstrated their potential applications in fog collection, oil-water separation, droplets' delivery, complex-flow distribution, visual biosensors, biomimetic tissue, adaptive drag control, and so on.

Although various SLIS have been demonstrated distinctive advantages and features under external stimuli challenges still remain in their practical applications. Firstly, some responsive SLIS are limited with complex, strict or high-cost preparation processes. Economical, reproducible, and effective fabrication to achieve well-organized micro/nanostructures and integrate stimuli-responsive components into SLIS is the first and foremost challenge. Secondly, reliability and durability of responsive SLIS are needed to improve. Besides substrate's long-term durability and mechanical stability, lubricant's depletion caused by cloaking effect or volatility should be paid attention (Carlson et al., 2013; Daniel et al., 2017). Appropriate selection of materials to avoid cloaking effect are advised. Choosing lubricants with higher viscosity and lower vapor pressure, or designing intelligent materials with self-healing or self-refill are also effective methods. On the other hand, since SLIS have potential applications in biomedical areas, including antibiofouling, antithrombosis, point-of-care diagnostics, surgery, and tissue integration, constructing responsive SLIS with biocompatibility materials and bioactive functions should be taken into consideration. Thirdly, smart SLIS mainly focused on introducing responsive components as solid substrate or lubricant, through stimuli responsive repellent liquid are still rare. More responsive repellent liquid should be developed. Furthermore, through design various responsive materials as different parts of SLIS at the same time, dual- or multi-stimuli responsive SLIS with multi-functionalities could be achieved. This may be a potential development direction for responsive SLIS.

## Author Contributions

YH, YZ, and XY proposed the manuscript. XY, YH, XZ, and JW wrote the manuscript. All authors revised the manuscript.

### Conflict of Interest

The authors declare that the research was conducted in the absence of any commercial or financial relationships that could be construed as a potential conflict of interest.
